# Supplementary material for: Fractionated Stereotactic Intensity-Modulated Radiotherapy for Large Brain Metastases: Comprehensive Analyses of Dose–Volume Predictors of Radiation-Induced Brain Necrosis
Source: Cancers (Basel). 2024 Sep 28;16(19):3327. doi: 10.3390/cancers16193327 (PMC11482639; doi:10.3390/cancers16193327)
Supplement: Supplementary file 1 [file cancers-16-03327-s001.zip › cancers-3175047-supplementary.pdf]

Supplementary Materials: Fractionated Stereotactic Intensity-Modulated Radiotherapy for Large Brain Metastases: Comprehensive Analyses of Dose-Volume Predictors of Radiation-Induced Brain Necrosis

Taro Murai, Yuki Kasai, Yuta Eguchi, Seiya Takano, Nozomi Kita, Akira Torii, Taiki Takaoka, Natsuo Tomita, Yuta Shibamoto and Akio Hiwatashi

Table S1. Logistic regression analyses of grade 1 or 2 brain necrosis in 5- and 10-fraction groups.

≥Grade 1 brain necrosis

	Odds ratio	(95% confidence interval)			*p*-value
UI	768	(0.06	-	9730000)	0.17
CI	0.46	(0.09	-	2.36)	0.35
PTV (cc)	1.00	(0.92	-	1.07)	0.88
V60GyE (cc)	1.08	(1.02	-	1.14)	0.01

	Odds ratio	(95% confidence interval)			*p*-value
UI	755	(0.05	-	10800000)	0.18
CI	0.46	(0.10	-	2.2)	0.33
PTV (cc)	0.99	(0.93	-	1.07)	0.86
V55GyE (cc)	1.06	(1.01	-	1.12)	0.02

	Odds ratio	(95% confidence interval)			*p*-value
UI	434	(0.03	-	6300000)	0.21
CI	0.48	(0.11	-	2.15)	0.34
PTV (cc)	1.00	(0.93	-	1.06)	0.88
V50GyE (cc)	1.05	(1.00	-	1.10)	0.04

≥Grade 2 brain necrosis

	Odds ratio	(95% confidence interval)			*p*-value
UI	704	(0.00	-	141000000)	0.29
CI	0.56	(0.07	-	4.21)	0.57
PTV (cc)	1.00	(0.92	-	1.09)	0.99
V60GyE (cc)	1.09	(1.02	-	1.17)	0.01

	Odds ratio	(95% confidence interval)			*p*-value
UI	802	(0.00	-	182000000)	0.29
CI	0.54	(0.08	-	3.76)	0.53
PTV (cc)	1.00	(0.92	-	1.09)	0.97
V55GyE (cc)	1.08	(1.02	-	1.14)	0.01

	Odds ratio	(95% confidence interval)			*p*-value
UI	440	(0.00	-	115000000)	0.34
CI	0.55	(0.09	-	3.48)	0.53
PTV (cc)	1.00	(0.93	-	1.08)	0.95
V50GyE (cc)	1.07	(1.01	-	1.13)	0.02

CI; conformity index PTV; planning target volume, UI; uniformity index, VxGyE; normal brain volume irradiated × Gy equivalent dose in 2 Gy/fraction.

Table S2. Brain necrosis incidence and irradiated normal brain volume.

		(mean ± SD)	Pt No	≥ Grade 1 (%)	≥ Grade 2 (%)
**V50EQD2 (cc)**	(<15)	4.4 ± 2.8	27	3 (11)	1 (4)
	(≥15, <30)	13.5 ± 2.2	23	1 (4)	1 (4)
	(≥30)	35.4 ± 15.3	28	6 (21)	5 (18)
**V55EQD2 (cc)**	(<15)	7.6 ± 4.1	34	3 (9)	1 (3)
	(≥15, <30)	21.3 ± 4.9	26	2 (8)	2 (8)
	(≥30)	48.1 ± 17.7	18	5 (28)	4 (22)
**V60EQD2 (cc)**	(<10)	8.1 ± 4.0	34	3 (9)	1 (3)
	(≥10, <20)	20.4 ± 3.8	20	1 (5)	1 (5)
	(≥20)	48.4 ± 21.6	24	6 (25)	5 (21)

Pt: patient, SD: standard deviation, VxEQD2; normal brain volume irradiated × Gy equivalent dose in 2 Gy/fraction.

Table S3. Logistic regression analyses of grade 1 brain necrosis in 5- and 10-fraction groups after categorizing variables.

		Odds ratio	(95% confidence interval)	*p*-value
PTV (cc)	(<8)	1.00		
	(≥8, <5)	0.35	(0.04 - 3.30)	0.36
	(≥15)	0.33	(0.05 - 2.40)	0.28
V60GyE (cc)	(<10)	1.00		
	(≥10, <20)	0.52	(0.05 - 5.61)	0.59
	(≥20)	5.55	(0.89 - 34.5)	0.07

		Odds ratio	(95% confidence interval)	*p*-value
PTV (cc)	(<8)	1.00		
	(≥8, <15)	0.41	(0.05 - 3.57)	0.42
	(≥15)	0.29	(0.03 - 2.56)	0.27
V55GyE (cc)	(<15)	1.00		
	(≥15, <30)	1.15	(0.16 - 8.11)	0.89
	(≥30)	8.18	(0.95 - 70.7)	0.06

		Odds ratio	(95% confidence interval)	*p*-value
PTV (cc)	(<8)	1.00		
	(≥8, <15)	0.52	(0.07 - 4.07)	0.53
	(≥15)	0.51	(0.08 - 3.38)	0.49
V50GyE (cc)	(<15)	1.00		
	(≥15, <30)	0.41	(0.04 - 4.33)	0.46
	(≥30)	2.97	(0.50 - 17.7)	0.23
